# The effect of match-play on acute post-match neuromuscular fatigue following Australian Football League Women’s (AFLW) competition

**DOI:** 10.5114/biolsport.2025.144412

**Published:** 2024-12-13

**Authors:** Emma Wilkinson, Tannath Scott, Matthew Green, Adam Hewitt, Mitchell Naughton

**Affiliations:** 1 School of Behavioral and Health Sciences, Australian Catholic University, Brisbane, Queensland, Australia; 2 School of Health Sciences and Social Work, Griffith University, Gold Coast, Queensland, Australia; 3 Brisbane Lions, Brisbane, Queensland, Australia; 4 School of Biomedical Sciences and Pharmacy, University of Newcastle, Callaghan, New South Wales, Australia; 5 Applied Sports Science and Exercise Testing Laboratory, University of Newcastle, Ourimbah, New South Wales, Australia

**Keywords:** Fatigue, Countermovement jump, External loads, Female physiology, Women’s sport

## Abstract

The Australian Football League Women’s (AFLW) is the premier national women’s competition in Australian Rules football. The aim of this exploratory study was to investigate the neuromuscular fatigue response to match-play and the external load correlates of this response in AFLW. Players (n = 22) wore a 10 Hz GNSS device and completed immediately pre- and post-match countermovement jumps (CMJ) on dual force plates for each match in the 2022 AFLW competitive season. Concentric, eccentric, and composite CMJ variables were selected a priori based on previously established validity, reliability, and sensitivity to detect neuromuscular fatigue. The change in each variable from pre- to post-match was analysed using linear mixed effect models and rank bi-serial correlation (*r*_bs_) effect size statistic. Linear mixed models were also constructed to examine the relationship between external load variables and the change in CMJ metrics. Each player was included as a random effect in these models. Match-play resulted in large negative effects to eccentric mean force, eccentric peak force, and force at zero velocity (all *r*_bs_ = 0.808 - 0.813), concentric impulse (*r*_bs_ = 0.646), flight time:contraction time (*r*_bs_ = 0.528), and jump height (*r*_bs_ = 0.491). Modelling identified high-speed running distance, repeated highintensity effort bouts, and acceleration load as significant (*p* < 0.05) correlates of the change in CMJ variables from pre- to post-match. The variance explained in these models was low (Conditional R^2^ = 0.128–0.186). Identified CMJ variables may be important to monitor fluctuations in neuromuscular fatigue, whilst external load variables may be useful in examining neuromuscular fatigue correlates in AFLW. Given the exploratory nature of this study, further research is necessary to explore these findings in a hypothesis driven framework.

## INTRODUCTION

Australian Football (AF) at the professional level is divided into the men’s Australian Football League (AFL) and the women’s Australian Football League (AFLW) competitions. In AF there are clear differences that exist between the genders in terms of match rules, external load profiles, and physiology. In the AFLW, 16 players compete across four 15-minute quarters in comparison to 18 players across four 20-minute quarters in the AFL [1]. Players in the AFLW are allowed 60 interchanges per match (as of 2023), while their male counterparts are restricted to 75 per match [1].^1^ Across competitive matches, AFLW players typically cover ~6 km per match while AFL players cover ~12 km per match [2]. Similarly, female players run ~370 m at a speed of ≥ 18 km · hr^−1^ in comparison to males who run substantially more distance of ~1800 m at the same threshold [2]. While these differences are potentially related to the discrepancies between match rules, they are also likely associated with other differences related to physiology (e.g., differences in muscle fibre type) [3], physical qualities (e.g., maximal running speed) [4], and body composition (e.g., lean soft tissue mass and fat mass) [5].

Professional AF at both the AFL and AFLW levels is a physically demanding team sport encompassed by locomotive-based external loads such as high-speed running, accelerations, and decelerations [1]. In the context of AF, external loads of training and match-play are quantified through objective monitoring of locomotor and collision actions through wearable microtechnology devices. These house global positioning/navigation systems (GPS/GNSS), accelerometers, and gyroscope sensors [6, 7]. The external loads of AF are typically quantified through metrics such as high-speed running (HSR), very highspeed running (VHSR), sprint distance, acceleration load, and total distance (TD) [1]. Recent findings have described the positional group differences in AFLW and identified high position specific running demands For example, midfielder-wingers have the highest average locomotor demands covering an average of 6.7 km per match at velocities up to 6.91 m · s^−1^.^1^ Whilst demands differ by position, overall players are required to have highly developed aerobic and anaerobic capacities, as well as technical abilities to maximise performance and negate the negative performance decrements associated with the accumulation of neuromuscular fatigue (NMF) [8].

The development of NMF occurs in response to repeated muscular contractions and can impede the production of muscular force and power output [9]. Fatigue, as a construct, contains several potential explanations and associated contributory factors. In the context of NMF, recent *in vivo* experimental research has reinforced that increased intramuscular inorganic phosphate (Pi) is a primary factor in ‘peripheral fatigue’ which specifies fatigue occurring downstream of the neuromuscular junction [10]. Further, muscle acidosis in response to intense exercise is likely to influence group III/IV muscle afferents which collectively contribute to fatigue originating from the central nervous system – termed ‘central fatigue’ [10]. Crucially, previous research has identified that females, compared to their male counterparts, are able to maintain a higher percentage of their relative force for longer and demonstrate a greater fatigue resistance when exercising at intensities above the critical metabolic threshold (equivalent to critical power or critical speed) [11]. Whilst not NMF *per se*, muscle damage from eccentrically-biased actions (e.g., accelerations/decelerations, change of directions, jumping etc.) as well as collisions (e.g., tackles) is also likely to contribute to changes in performance metrics observed in the days following matchplay [12]. Finally, differences in sex hormones (e.g., estrogen production) may also modulate the muscle damage response [13].

There are significant increases in NMF during AFL match-play, which can likely be attributed to the external loads experienced by players [14, 15]. The presence of NMF has shown to impede relative high-intensity running output (m · min^−1^) during AFL match-play which is considered a key performance indicator [8, 16]. This response has prompted sport scientists to monitor NMF in the limited recovery window (~5–7 days) between competitive matches. Given the practicality, time efficient nature, and simplicity, the countermovement jump (CMJ) undertaken on force plate technology has been described as the practical reference standard to measure NMF in elite AF [14]. This protocol has shown to produce highly valid and reliable metrics with which to quantify post-match NMF [17]. Among the many available CMJ metrics, the flight time to contraction time ration (FT:CT) and peak velocity have shown to be the most sensitive to detect practically meaningful changes following AFL match-play [18, 19]. Importantly in the context of the previously mentioned physiological differences between males and females and AFL and AFLW, the characterisation of NMF has yet to be explored in AFLW. Further, the external load correlates of NMF in AFLW are not known. Such knowledge could assist with training prescription in allowing fitness staff to tailor exposures to develop resilience and athlete capacity to tolerate NMF.

The current evidence suggests that the NMF response of female AFLW players may differ to that of male AFL players. However, this has yet to be investigated in the context of AFLW and post-match NMF. The objectives of this study are therefore to firstly examine the CMJ metrics are the most sensitive to detect changes in NMF and, secondly, to establish which AFLW match-related external loads have the greatest association with NMF. This study is undertaken in an exploratory and hypothesis generating, rather than hypothesis confirmatory framework.

## MATERIALS AND METHODS

### Subjects

Twenty-two injury-free professional AFLW players (mean + SD age; 25.0 + 3.7 years) from a single AFLW club participated in this study. Informed consent was obtained from each participant and ethical approval was granted from the university research ethics committee (2023-3266N) in correspondence to the *Code of Ethics of the World Medical Association* (Declaration of Helsinki). To be eligible to participate in this study, each player must have been injury free prior to match-play nor did they sustain one during the match, played ≥ 4 matches, and played > 10 minutes of the match.

### Design

The study adopted a within-subject repeated-measures longitudinal observational design with a quantitative approach and a retrospective analysis of the 2022 AFLW season (*n* = 13 games). The matchrelated external loads quantified using microtechnology were the independent variables, while the CMJ metrics measured on ForceDecks (ForceDecks, Vald Performance, Australia) were the dependent variables.

### Methodology

The data collection occurred within the 2022 AFLW (Season 7) inseason period. Following a standardised warm-up protocol, each player performed a maximal CMJ without arm-swing in triplicate using the previously validated portable dual force plate system (ForceDecks, Vald Performance, Australia) between 120- and 60-min pre-match, and by 30-min post-match. The post-match jumps were undertaken shortly after the match to ensure post-match fatigue was captured prior to players undertaking their recovery interventions, media commitments, and travel arrangements. Due to the potentially large number of CMJ variables, the included metrics were selected *a priori* to the analysis based on their established validity, reliability, and sensitivity in previous studies of team sport cohorts [18, 20]. These variables were jump height (impulse-momentum method) (cm), contraction time (ms), peak power (W), stiffness (N · m^−1^), reactive strength index modified (RSI_mod_) (m · s^−1^), FT:CT, concentric peak velocity (m · s^−1^), concentric impulse (Ns), concentric mean force (N), eccentric mean force (N), force at zero velocity (N), eccentric duration (ms), eccentric peak force (N), and countermovement depth (cm). Most metrics examined have excellent validity when captured on the ForceDecks system (< 5% difference to a lab-based inground forceplate system) [36]. Tests were undertaken using the manufacturer’s suggested countermovement jump test protocol [36]. The best jump of the three trials (based on jump height) was included in the analysis.

Throughout match-play each player wore a designated microtechnology device (Catapult Vector S7, Catapult Sports, Melbourne, Australia) featuring a 10 Hz GNSS sensor via an in-built pocket on their playing jersey positioned on the thoracic region of their spine. These were turned on and positioned 10-minutes prior to the pre-match warm up. Immediately post-match each device was collected, and data was downloaded to Openfield (Catapult Sports, Melbourne, Victoria) for warehousing. The external load variables which were included in this study are presented in Table 1. The select external load variables included repeated high intensity effort (RHIE) total bouts, HSR distance, and acceleration load. RHIE is typically described as ≥ 3 high intensity efforts such as sprints, accelerations or tackles performed within 21 seconds of each other [21]. Acceleration load is defined as the amount of speed change activity and is calculated by the accumulation of all absolute acceleration and deceleration values [22]. Microtechnology-related quality variables including average horizontal dilution of precision (HDOP), GNSS quality, and satellite count were also quantified.

**TABLE 1:** External loads for Women’s Australian Rules football (AFLW) grouped by position (mean ± SD).

	Back	Forward	Midfield	Ruck	Wing
**Absolute**					
Field time (min)	56.3 ± 8.5	51.4 ± 9.5	56.7 ± 5.2	55.6 ± 4.6	55.0 ± 3.8
Total distance (m)	6822 ± 1079	6031 ± 1066	7380 ± 827	6660 ± 596	7568 ± 586
Total PlayerLoad (AU)	678 ± 116	591 ± 135	835 ± 132	631 ± 61	758 ± 58
Maximum velocity (m/sec^−1^)	7.03 ± 0.50	7.00 ± 0.53	6.86 ± 0.50	6.83 ± 0.33	7.22 ± 0.36
RHIE bouts (no.)	18 ± 6	19 ± 5	24 ± 5	17 ± 5	25 ± 6
Acceleration load (AU)	1609 ± 276	1476 ± 276	1737 ± 211	1496 ± 116	1658 ± 177
HSR distance (m)	771 ± 285	733 ± 272	710 ± 184	442 ± 131	1164 ± 362
VHSR distance (m)	178 ± 114	162 ± 116	106 ± 80	81 ± 25	245 ± 98

**Relative**					
PlayerLoad (AU/min^−1^)	12.1 ± 1.5	11.6 ± 2.1	14.8 ± 1.9	11.4 ± 0.7	13.8 ± 0.9
Distance (m/min^−1^)	121.4 ± 7.6	118.4 ± 11.4	130.3 ± 7.7	120.1 ± 4.8	137.9 ± 7.7
HSR (m/min^−1^)	28.9 ± 6.7	31.5 ± 9.0	33.1 ± 7.3	18.9 ± 4.8	44.0 ± 10.3

Note. Arbitrary units (AU); high-speed running (HSR; > 4.7 m · s^−1^), repeated high intensity efforts (RHIE); very high-speed running (VHSR; > 5.8 m · s^−1^).

### Statistical Analysis

This study used an observational and repeated-measure longitudinal design. The approach to the analysis was considered through an exploratory rather than hypothesis confirmatory lens. The open access JASP (Version 0.18) software and RStudio (Version 2023.09.0+463) were used for analysis. Firstly, visual inspection of CMJ variable residual histogram and Q-Q plots was undertaken to check the normality assumption. Thereafter, CMJ variables were analysed using linear mixed effects models using the *lme4* package with the post-match CMJ variable as the outcome, the pre-match CMJ variable as the fixed effect predictor, and the player ID as a random effect. Following this, as the variables were mostly non-parametric, a rank bi-serial correlation (*r*_bs_) effect size measure was selected, and 95% confidence intervals (CI) for this effect were calculated and included. The interpretation for these effects were based on Pearson’s *r* correlations, with small effect size *r* = 0.1–0.3, medium effect size *r* = 0.3–0.5, and large effect size *r* ≥ 0.5 [23]. Finally, to examine the correlates of NMF, linear mixed models were constructed between each candidate predictor (external load variable) and response (change in CMJ metric from pre- to post-match) variable with each individual player ID included as a random effect. Both the variance explained only accounting for the fixed effects (marginal R^2^), and the variance explained by accounting for both the fixed and random effects (conditional R^2^) were examined. This analysis was performed using the *lme4* and *performance* packages with models developed for all the select external load and CMJ variables.

## RESULTS

The external loads for each positional group analysed across the 2022 AFLW season are presented in Table 1. Across all player match observations, average HDOP was 0.72 (± 0.08), while satellite count was 13.7 (± 1.8) and GNSS quality was 63.3% (± 5.5%).

The change in NMF broadly categorised as composite, concentric, or eccentric CMJ variables [20] is presented in Table 2. Table 2 also presents the pre- and post-match (mean ± standard deviation [SD]) measures, the mean difference, the fixed effect *p*-value, the *r*_bs_ effect size, and 95% upper and lower confidence intervals.

**TABLE 2:** Pre- and post-match (mean ± SD) countermovement (CMJ) metrics and fixed effect results from linear mixed effect modelling for the included variables which are categorised as composite, concentric, or eccentric.

Variable	Pre (SD)	Post (SD)	Mean Difference	*p* value	Rank bi-serial (rbs) correlation	rbs Lower 95% CI	rbs Upper 95% CI
**Composite**							
Jump Height (Impulse-Momentum) (cm)	33.2 (5.6)	31.8 (5.9)	-1.4	< .001	0.491	0.360	0.604
Contraction time (ms)	808 (105)	844 (111)	36	< .001	-0.555	-0.657	-0.433
Peak Power (W)	3330 (348)	3292 (352)	-38	< .001	0.154	-0.005	0.305
Stiffness (N.m^−1^)	5126 (1355)	5470 (1502)	344	< .001	-0.426	-0.546	-0.284
RSI_mod_ (m · s^−1^)	0.475 (0.093)	0.441 (0.097)	-0.034	< .001	0.551	0.426	0.655
Flight time: contraction time ratio	0.721 (0.103)	0.686 (0.105)	-0.035	< .001	0.528	0.399	0.637

**Concentric**							
Concentric Peak Velocity (m · s^−1^)	2.7 (0.2)	2.6 (0.2)	-0.1	< .001	0.449	0.309	0.569
Concentric impulse (Ns)	171 (16)	166 (16)	-5	< .001	0.646	0.543	0.730
Concentric mean force (N)	1299 (143)	1275 (142)	-24	< .001	0.452	0.316	0.570

**Eccentric**							
Eccentric mean force (N)	665 (79)	660 (79)	-5	< .001	0.808	0.743	0.858
Force at Zero Velocity (N)	1580 (210)	1459 (213)	-121	< .001	0.813	0.750	0.860
Eccentric duration (ms)	524 (77)	560 (90)	36	< .001	-0.634	-0.721	-0.528
Eccentric peak force (N)	1589 (214)	1465 (216)	-124	< .001	0.810	0.747	0.859
Countermovement depth (cm)	-32.0 (5.1)	-29.4 (5.8)	2.6	< .001	-0.754	-0.816	-0.676

Note. Relative strength index (RSI).

All CMJ variables were statistically significant (*p* < 0.001), indicating a difference from pre- to post-match. Eccentric mean force, peak power (PP), force at zero velocity, eccentric duration, eccentric peak force, CMJ depth, concentric impulse, FT:CT, RSI_mod_, and contraction time were all considered large effects (*r*_bs_≥ 0.5). Jump height, concentric peak velocity, and concentric mean force were all considered with moderate, statistically significant effects (*r*_bs_= 0.3–0.5). PP was the only variable with a small effect size (*r*_bs_< 0.3). The external load variables that were significant (*p <* 0.05) predictors of the select post-match NMF variables are presented in Table 3. The RHIE total bouts, HSR distance (> 4.7 m · s^−1^), and acceleration load were statistically significant predictors of the post-match change in contraction time (m · s^−1^), eccentric duration (m · s^−1^), and countermovement jump depth (cm) (*p* < 0.05). The variance explained by conditioning the analysis on the random effect of the individual player responses (Conditional R^2^) increased the variance explained compared to just the fixed effects (Marginal R^2^) in the models, but the variance explained was low (Conditional R^2^ = 0.128–0.186) in those models which were significant (*p* < 0.05) (Table 3).

**TABLE 3:** External load correlates of post-match neuromuscular fatigue (NMF).

Predictor variable	Response variable (change)	Marginal R2	Conditional R2	p value
RHIE total bouts (no.)	Contraction time (ms)	0.041	0.186	0.0178
HSR distance (m)	Contraction time (ms)	0.038	0.157	0.0278
RHIE total bouts (no.)	Eccentric duration (ms)	0.034	0.147	0.0281
HSR distance (m)	Eccentric duration (ms)	0.034	0.128	0.0338
Acceleration load (AU)	Countermovement jump depth (cm)	0.035	0.180	0.0373
RHIE total bouts (no.)	Countermovement jump depth (cm)	0.030	0.178	0.0460

*Note*. Repeated high intensity efforts (RHIE); high-speed running (HSR).

## DISCUSSION

This is the first study to characterise the NMF response to AFLW match-play. This project primarily aimed to determine the most sensitive CMJ variables to monitor NMF post-match in AFLW and examine which external load variables have the largest association with NMF in AFLW. Regarding the first objective, most of the examined NMF variables are sensitive to change in NMF status in the acute (< 2 hour) post-match window. This suggests that in this period, players are significantly fatigued, and this is evidenced by large effects of match-play on composite, concentric, and eccentric-biased CMJ variables. These included jump height, a variable which is arguably easier for coaches to measure, as well as concentric and eccentrically focused variables which require the use of force plates. Previous findings have demonstrated that NMF impedes physical capacity and match exercise intensity in professional male AF which negatively impacts external load performance indicators [8, 16]. Hence, monitoring NMF and recovery between AFLW matches is imperative to optimise athletic performance and the variables identified in our findings could therefore be used to monitor NMF in AFLW.

For the second objective, several variables act as significant predictors of the change CMJ variables across composite, concentric, and eccentric-biased CMJ variables. Although, it is important to highlight that the variance explained in these changes was relatively low, even after accounting for the individual nature of the post-match NMF response in a fixed and random effect model framework. These predictor variables may therefore be important to monitor when monitoring the change in external loads throughout a season. Importantly, the selection of CMJ variables analysed were based on the findings of previous literature as to potential variables of importance [18]. Although, it should be noted that given the scarcity of similar literature in AF, most of the variables selected were based off the sensitivity, validity, and reliability found in other sports (e.g., professional rugby union) and in the male context (e.g., Howarth et al., [20]). This highlights the lack of research available in the female athlete space. Indeed, increasing the representation of women in sports science research, as both participants and researchers, has recently been brought into sharp focus [24, 25].

Analysis of the NMF data, highlights the significant increase in eccentric duration and decreases in eccentric peak and mean force. This indicates that players spent a longer duration developing force during the eccentric (i.e., braking) phase of the CMJ movement to ultimately produce less force eccentrically. Coupled with this was an increased limitation in range of motion (as evidenced by a decrease in the CMJ depth) and an increase in contraction time. This is likely a result of the repetitive use of the eccentric component of the stretch shortening cycle (SSC) during match-play (e.g., through accelerations, change of directions) leading to damage of the skeletal muscle ultrastructural components, a process known as exercise induced muscle damage (EIMD) [26]. Although the mechanisms for EIMD have not been resolved, the current literature suggests that experiencing particularly large amounts of unaccustomed eccentric exercise causes primary muscle damage through ‘popped sarcomeres’ and secondary inflammation of the muscle fibers leading to short-term performance decrements measured through force production [26]. Hence, the observed decrements in eccentric force production may be attributed to the mechanisms responsible for EIMD.

A significantly large decrement of RSI_mod_ and FT:CT was also observed from pre- to post-match. These metrics describe the time spent in the air relative to the time spent on the ground in both the eccentric and concentric phases and are commonly utilised for NMF monitoring and quantifying adaptation [27]. This observation suggests that players altered their movement strategy by spending more time developing force as opposed to their duration in the air. This is likely related to the large decrements observed in eccentrically biased metrics, namely eccentric mean force, force at zero velocity, and eccentric peak force and increases in eccentric duration. These findings suggest that a player’s ability to develop force eccentrically is hindered following the external loads experienced during matchplay. Therefore, a change in movement strategy is necessary. Supporting findings in professional male AF found that FT:CT is the most sensitive CMJ variable to detect practically meaningful changes in NMF [27]. Additional findings in elite female rugby union also support the use of FT:CT to track acute NMF [28]. One suggestion for the explanation for impaired FT:CT has been a decrease in muscle tendon stiffness after SSC exercise which is correlated to an increase in ground contact time in drop jump performance [29].

The change in PP from pre- to post match was observed to be significantly lower post-match which align with findings of previous research which demonstrated that PP via a cycle ergometer test can detect significant changes in NMF following elite junior AF matchplay [14]. There were, however, some differences in the magnitude of the effect in the prior study and those in our analysis. The researchers attribute this discrepancy to the cycle ergometer’s specific quantification of the concentric component of force production, which may be overlooked by the CMJ protocol which quantifies concentric and eccentric forces [15]. While the eccentric metrics appear to be the most dominant of the sensitive metrics, we found that concentric impulse, concentric peak velocity, and concentric mean force are also highly sensitive measures to detect post-match NMF. This finding suggests that the ability of AFLW players to develop concentric force rapidly is also hindered post-match. The discrepancy between our findings and that of previous findings (e.g., Wehbe et al., [15], may be attributed to the differences in age, sex, and body composition between athlete participants, and how these differences might manifest to those observed in previous literature [5].

This project also aimed to explore the external load variables that are the largest correlates of NMF in AFLW. In analysing the external load variables in the current study, volume-based metrics such as total distance (m), high speed running distance (m), and total PlayerLoad (AU) were higher than previously reported external loads in AFLW, while the relative metrics such as distance (m · min^−1^), and relative PlayerLoad (AU/min) were similar.^1^ For example, Clarke et al., [1] reported midfielders run 5813 m of total distance on average per match, at a relative speed of 128.4 m · min^−1^, while in the present study midfielders ran 7380 m of total distance at relative speed of 130.3 m · min^−1^. Further analysis suggests that the increase in game duration (min) from 45.7 min on average for midfielders [1], as an example, to 56.7 min in the present study has contributed to these differences in absolute volume-based external load variables. Longitudinal research by Clarke et al., [35] supports this suggestion in some positional groups with an increase in game duration across seasons for midfielders and forwards, but not for defenders and backs. In a sport with limited rotations like AFLW, this suggests that backs and defenders are more likely to be rotated than midfielders or forward positional players, which could indicate a high perceived level of importance to these positional groups by the coaching staff.

It is important to recognise that our study is exploratory and hypothesis generating in nature. Further hypothesis-led confirmatory research is required to determine a causal relationship between the select external load and NMF measures. The current findings suggest that there are several external load variables which act as significant but weak predictors to post-match NMF. For example, both RHIE total bouts and HSR distance (> 4.7 m · s^−1^) are related to an increase in CMJ eccentric duration. The findings here should be interpreted with due caution. This, however, suggests that HSR (i.e., sprinting), and repetitions of high intensity efforts may be partially responsible for prolonged eccentric-phase durations to produce less eccentric force. This is likely related to the repeated use of the SSC during match-play resulting in metabolic disturbances and impaired excitation–contraction coupling which has been described in previous research [30]. Further, VHSR (> 5 m · s^−1^) has been previously shown to be largely correlated to decrements in CMJ PP, with larger VHSR distances exacerbating the response [31]. Indeed, Hader et al., [31] observed that VHSR distance may be responsible for up to ~50% of post-match NMF in soccer. This is thought to be related to the high eccentric muscle activity required during the swing phase of running gate. Hence, it is no surprise that RHIE and HSR distance is related to a reduced ability to develop force eccentrically post-match in our findings. Thus, it would appear important for players to have a high ability to tolerate such loads to improve postmatch recovery. One important observation made by Hader et al., [31] and other studies [12], is that they have identified PP as being sensitive to NMF post-match but at different time points to our study (e.g., 24-, 48-, and 72-hours post-match). Thus, further research would be necessary to include further testing time points to characterise the full-time course of post-match NMF. This would also be required to confirm our findings and further explore the thresholds for RHIE and relative velocity bands in AFLW.

Acceleration load was found to be related to reduction in CMJ depth, albeit with a low variance explained. Previous research in professional male AF has observed associations between increased acceleration load with increased levels of creatine kinase (CK), a biomarker of muscle damage [32, 33]. It is important to note that NMF and muscle damage are independent of each other, although, there are acute situations where muscle damage may influence fatigue. In our novel study, muscle damage may have been present immediately post-match but the time course of CK changes would suggest that any muscle damage peaks in the days (not hours) following the match [12]. Research in professional soccer also supports this notion, having found an association between the frequency of hard accelerations, and increases in post-match NMF [34]. Our findings could be related to the heavy recruitment of the active and passive neuromuscular components through repeated SSC exercise to perform acceleration/deceleration movements resulting in decrements in eccentric force production, which were observed in our CMJ findings. This decreased ability to produce force eccentrically potentially has implications on the athlete’s range of motion and hence, the depth achieved during the lowering phase of the CMJ. To our knowledge, this is the first study to explore these associations with NMF in women’s AF. Importantly, many of the variables examined had no significant relationship with the change in performance variables, an observation that requires further examination. Given the exploratory nature of these findings, further research must be conducted to draw valid and reliable inferences as to the exact nature of the relationship between the variables which were examined. Indeed, exploring whether higher strength and fitness are protective against NMF in AFLW could be an important future avenue of research.

There are limitations present in this study, other than those already acknowledged. The measurement of NMF was only undertaken immediately (i.e., within 2-hours) pre- and post-match. This may have had an impact on the variability within the data, as for some athletes fatigue may have yet to be fully apparent. Ideally this collection time would be further standardised in subsequent research (e.g., 1 hr pre and 1 hr post), and the full-time course of NMF would be documented including these time points but through to 24-, 48-, and 96-hours post-match to understand the sensitivity of CMJ metrics for prolonged NMF. Indeed, the NMF metrics are known to fluctuate through the acute, residual, and persistent fatigue recovery periods.^12^ Further, our sample of 22 participants measured over one AFLW season may impact the generalisability of our findings to players outside of our sample. Finally, as we did not quantify the proportion of the players who were oral contraceptive users or who were undergoing different phases of their menstrual cycle, we cannot discount the influence of this on the findings. Future research seeking more generalisable findings should utilise a larger sample size of players and teams across multiple AFLW seasons.

## CONCLUSIONS

The findings of this study may be applied in AFLW and similar female team sports such as rugby league, rugby sevens and soccer. The CMJ protocol described in this study is a useful tool to monitor changes in NMF status in team sport players. Amongst the variables, RSI_mod_, eccentric mean force, force at zero velocity, eccentric peak force, and eccentric duration can detect practically meaningful changes in NMF. These CMJ metrics may be utilised to provide coaches and support staff working in elite female team sports with important insight their athlete’s post-match recovery. The findings may not be generalisable to sub-elite and recreational players due to the differences regarding the external loads experienced and the capacities of the athletes which exist between populations.

Of the external load variables, RHIE total bouts, HSR distance, and acceleration load were the identified contributors to NMF in AFLW. For practitioners considering which variables are important to monitor within their matches, these metrics might provide a useful starting point with which to do so. These are exploratory findings and warrant exploration in future research. Coaches and support staff may benefit from these findings to impose NMF related external loads in pre-season training sessions to promote adaptation and improve athlete ability to tolerate such loads which may in part assist in reducing the accumulation of post-match NMF during the in-season period.

This study is the first to characterise the NMF response to AFLW match-play. Further, this is the first study to explore the effect of external loads on NMF and associated CMJ metrics that are most sensitive to detect changes in NMF in AFLW. The findings suggest that metrics that incorporate an eccentric component, a time component, or a strategy component are sensitive to detect post-match NMF.

This may be useful to practitioners working in AFLW to monitor postmatch NMF and recovery more accurately, however caution should be exercised not to generalise the findings too broadly, in light of the exploratory nature of the present study. External loads that maximally recruit the SSC appear to be the most indicative of post-match NMF, and these may be useful to monitor external loads in AFLW. Further research is required to confirm these findings and given the weak relationships identified, these should be interpreted with caution.

## Data Availability

The anonymised data that support the findings of this study are available from the corresponding author upon reasonable request.

## HIGHLIGHTS

– The study demonstrated that countermovement jump (CMJ) metrics, especially those emphasising eccentric mean and peak force, concentric impulse, and flight time:contraction time are sensitive indicators of neuromuscular fatigue (NMF) in AFLW players post-match.
– External load factors, such as repeated high-intensity effort bouts and high-speed running distance were identified as significant but weak predictors of post-match NMF changes in AFLW players..
– This exploratory study is the first of its kind in characterising NMF in AFLW, suggesting that specific CMJ metrics and external load variables can inform tailored fatigue monitoring practices in AFLW players. Given the exploratory nature of the analysis, further hypothesis confirming research is necessary to examine the causal relationship between match-play and fatigue in AFLW.
